# Successfully drug treatment for necrotizing fasciitis caused by *Streptococcus pyogenes* in 88‐year‐old patient

**DOI:** 10.1002/ccr3.7633

**Published:** 2023-07-06

**Authors:** Nadia Ladjouzi, Jihène Ben Hassen, Idir Mebarki, Mohamad Al Zoabi, Adel Alaysh, Joël Schlatter

**Affiliations:** ^1^ Médecine gériatrique aigue Hôpital Paul Doumer, Assistance Publique des Hôpitaux de Paris Paris France; ^2^ Pharmacie, Hôpital Paul Doumer Assistance Publique des Hôpitaux de Paris Paris France

**Keywords:** antibiotics, group A *Streptococcus*, necrotizing fasciitis, older adult

## Abstract

Necrotizing fasciitis (NF) is a rare soft‐tissue infection generally treated by emergency surgical intervention. We report a case of successfully drug treatment for NF in older patient with comorbidities thus avoiding surgical intervention.

## Graphical abstract

Severe edema with bullous lesions.

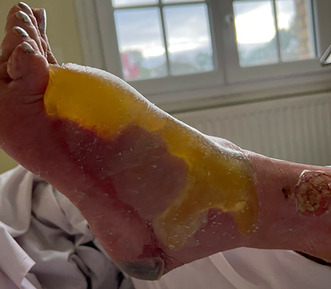

## 1 INTRODUCTION

Necrotizing fasciitis (NF) is a soft‐tissue infection that is rare but rapidly progressive with incidence estimated to be 4.0 to 15.5 cases per 100,000 population.^1^ NF is characterized by a progressive destruction of the muscle fascia and overlying subcutaneous fat. Due to a high risk of mortality, prompt diagnosis of signs and symptoms and treatment are essential to avoid an adverse patient care pathway including sepsis, amputation, and death.^2^ Monomicrobial NF is most commonly associated with Gram‐positive organisms such as group A *Streptococcus*.^1^ Pyrogenic toxics produced by this germ can induced streptococcal toxic shock syndrome associated with worse prognosis particularly in elderly population.^3^ In this report, we present a case of lower‐extremity NF caused by *Streptococcus pyogenes* that was successfully treated without interval surgical intervention.

## 2 CASE REPORT

**FIGURE 1:**
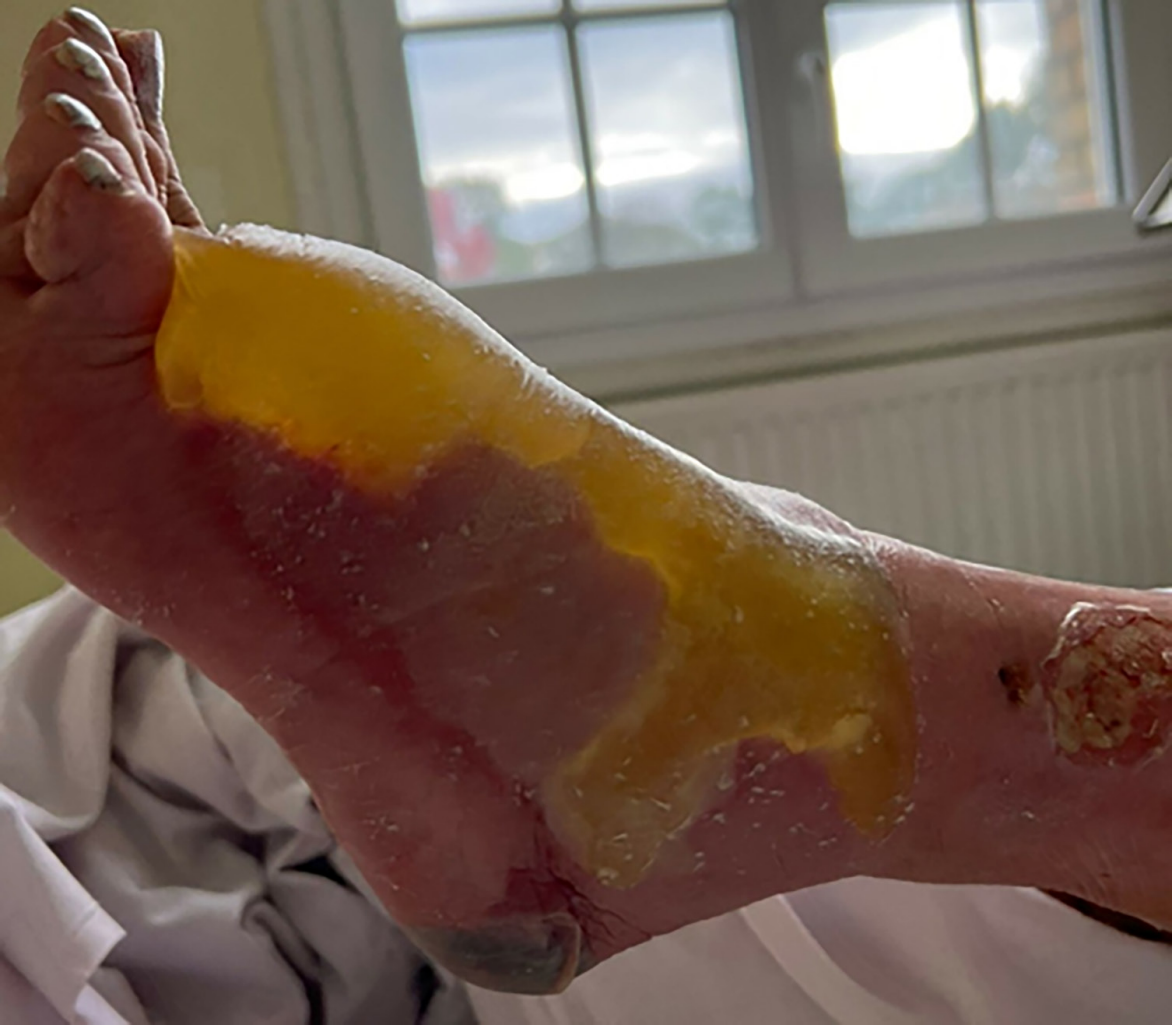
Aspects of the forearm exhibiting significant edema with bullous lesions at the time of presentation.

**FIGURE 2:**
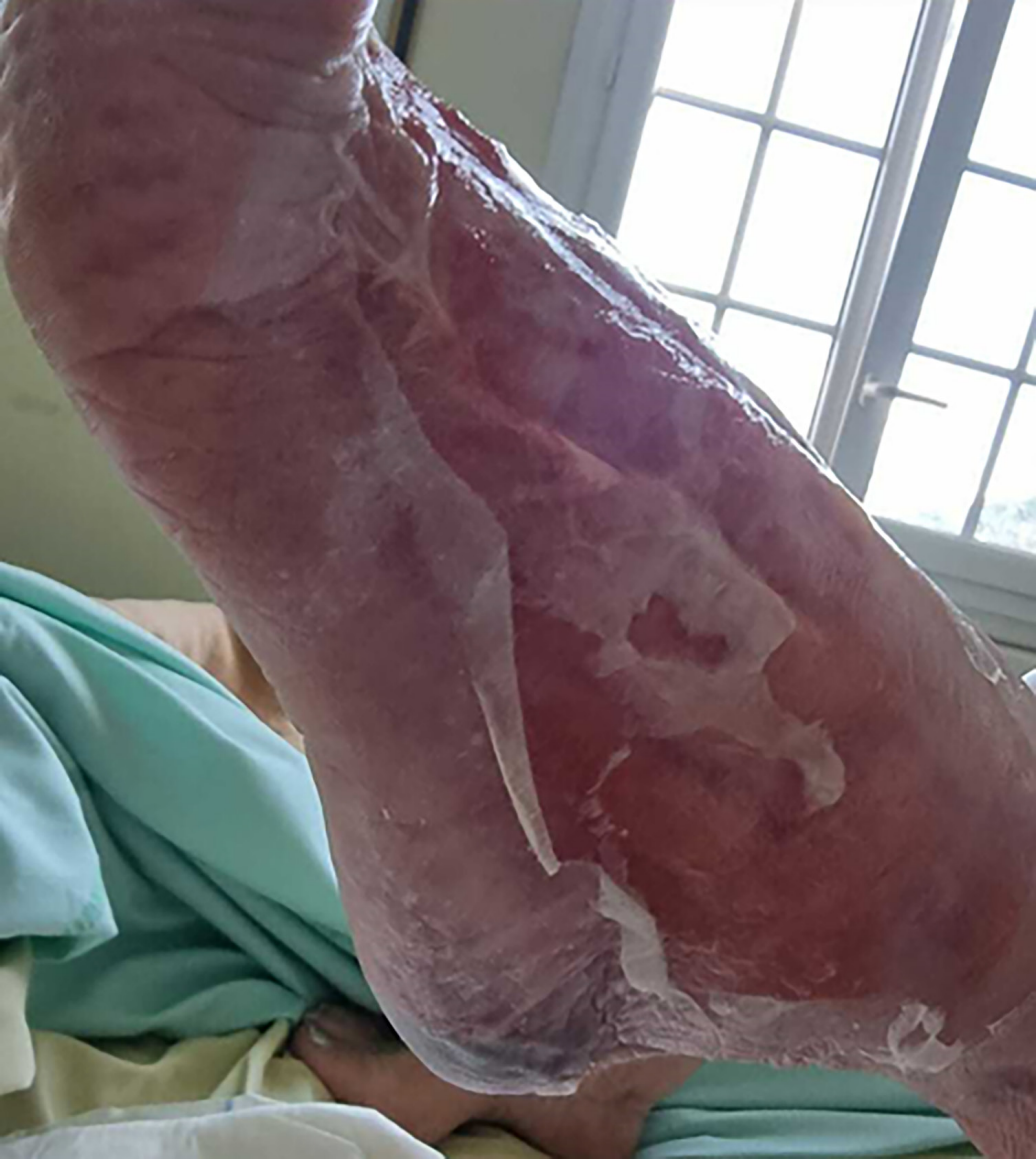
Aspects of the forearm exhibiting significant healing of NF lesions at Day 15.

A 88‐year‐old woman, 148 cm tall, weighing 63.2 kg, with history of hypertension, epilepsy, diabetes, and transient ischemic attack, presented to the emergency department with left lower leg edema. She had no other significant medical history. The patient complained of left lower leg pain and a red patch 1 day before hospitalization. She suffered an infected scar on the left tibia for 15 days associated with asthenia and general weathering, making her unable to stand. Neurological examination has been normal. A diagnosis of erysipelas was established in the emergency department, and an empiric drug therapy was initiated with the combination of amoxicillin and clavulanic acid. The patient was then referred to our hospital in acute geriatric medicine. On initial evaluation, the leg is inflammatory and painful in a febrile setting, with an entry site on the anterior surface of the left tibia. There was a 3 cm wound with a necrosis on the left heel (Figure 1). The patient was calm and coherent. The quick Sequential Organ Failure Assessment (qSOFA), an effective tool to assess the mortality risk of septic shock, was estimated to 1 considering the patient's altered mental state. Physical examination revealed the following: temperature = 37.8°C, blood pressure = 130/62 mmHg, heart rate = 72 beats/min, and oxygen saturation = 93% on room air. Laboratory test were significant with white blood cell count of 16.99 g/L (reference range, 3.77–9.05 g/L), hemoglobin of 11.0 × 10 g/L (reference range, 11.8–15.0 × 10 g/L), hematocrit of 33.3% (reference range, 34.9–44.6%), elevated absolute neutrophil count of 15.51 g/L (reference range, 1.90–5.72 g/L), blood potassium of 2.62 mmol/L (reference range, 3.40–4.50 mmol/L), glucose of 1.27 g/L (reference range 4.56–6.38 g/L), and elevated C‐reactive protein (CRP) of 303.7 mg/L (reference range, 0.5–5.0 mg/L). The blood cultures exhibited the gram‐positive pathogen *Streptococcus pyogenes*. On hospital Day 1, amoxicillin at 8 g/d was introduced instead of amoxicillin‐clavulanic acid and clindamycin 1200 mg/d was added for a 15‐days period because of the frailty situation (diabetes). In addition, a preventive enoxaparin 4000 IU/d anticoagulant treatment was initiated to limit the risk of thromboembolism. At Day 2, a computerized tomography (CT) angiography of the upper limbs and an expert advice from a plastic surgeon have been ordered, since our hospital is not fully equipped for this procedure. The CT angiography illustrated a pre‐occlusive stenosis of the mesenteric artery estimated to be 90% based on NASCET (North American Symptomatic Carotid Endarterectomy Trial) criteria. Moreover, it showed infiltration of the soft tissues of both lower limbs, with cellulitis, fasciitis and myositis in the left ankle and foot. The diagnostic of NF was established by clinical, biological, and radiologic findings such as the diabetic field, the fast progression of necrosis, the appearance of bubbles and crackles within 24 hours, the inflammatory syndrome, and the deep fasciitis damage. Despite patients with high‐grade stenosis (NASCET 70 to 99 percent) should be considered for referral to surgeons, the plastic surgeon was not considered the revascularization surgery as emergency because of the frailty of the patient and the extent of damage. In combination with antibiotic dual therapy, dressings were made every day with soap and water to remove fibrin, drying and then applying alginate‐based dressings. Rapidly, the evolution of the NF was favorable with reduced inflammatory signs and redness, and a lack of fibrin and suppuration. At Day 15, the clinical and biological evolution was positive with a significant decrease of white blood cell count of 6.10 g/L and CRP of 9.6 mg/L. Healing of the NF lesions progressed in a positive way (Figure 2). The subsequent course of the patient was favorable, and she was transferred to her house. A geriatrician in outpatient care followed the patient.

## 3 DISCUSSION

Type II NF associated with group A Streptococcus are responsible for some clinical presentations including toxic shock syndrome.^4^ The potential for aggressive NF is related to the number of exotoxins produced by the bacteria and several features of cell surfaces molecules known as M‐proteins.^5^ In consequence, the inflammatory response is due by the production of cytokines especially interleukin 1, interleukin 6, and tumor necrosis factor‐α leading to the disastrous shock with NF. One result is systemic thrombosis of blood vessels, which prevents the host's immune system combatting the infection and induces necrotic tissue. The typical patient of type II NF is quite young and fit, with a previous history of skin injury, commonly from extremity trauma.^5^ Appropriate treatment for NF is the surgical exploration and debridement of the affected tissue remaining the mainstay of therapy, which sometimes requires limb amputation.^6^ The most recent Infectious Disease Society of America guideline recommends penicillin plus clindamycin to treat documented group A Streptococcus infections.^7^ The patient in this study presented risk factors of severe disease related to age and diabetes in particular. The surgical debridement was not performed but treatment with amoxicillin‐clavulanic acid was administered as empiric measure at the time of admission of emergency department. However, this initiated treatment failed to comply with the recommendations.^6^ As the causative bacterium of NF was group A Streptococcus, the treatment on arrival of the patient was promptly reconsidered with a combination of amoxicillin‐clavulanic acid and clindamycin. The use of clindamycin in the patient was justified by its potent inhibition of virulence protein synthesis in bacteria and its ability to inhibit GAS phagocytosis by neutrophils.^5^, ^8^ The immediate empirical administration of broad‐spectrum antibiotics in patients suspected NF is justified based on the microbiological classification of the disease. The initial treatment should comprise antibiotics targeting aerobic gram‐positive cocci, gram‐negative rods, and some anaerobes. This treatment lust be readjusted according the results of tissue cultures and sensitivity patterns.^9^ Additionally, wound care with hydrogen fiber dressing made of woven fibers of sodium methylcellulose was used to absorb exudate forming a cohesive gel. This dressing was non‐adhesive and can be easily removed. It was replaced less often, and do not require analgesia for dressing change.^10^ Another advantage of the hydrofiber dressing is that bacteria adhere to the dressing fiber, potentially reducing cross‐infection.^11^

## 4 CONCLUSION

Here, we reported a drug treatment for NF in older patient resulted in a favorable outcome despite her advanced age and comorbidities. Proactive antibiotic treatment for NF is a high priority and could prevent emergency surgical debridement in combination of correct dressing approach.

## AUTHOR CONTRIBUTIONS

**Nadia Ladjouzi:** Validation; visualization. **Jihène Ben Hassen:** Conceptualization; funding acquisition; validation; visualization. **Idir Mebarki:** Validation; visualization. **Mohamed Al Zoabi:** Validation; visualization. **Adel Alayash:** Funding acquisition; validation; visualization. **Joël Schlatter:** Conceptualization; funding acquisition; validation; writing – original draft.

## FUNDING INFORMATION

None of the authors has any financial disclosures.

## CONFLICT OF INTEREST STATEMENT

The authors declare that there is no conflict of interests regarding the publication of this paper.

## ETHICS STATEMENT

Ethics approval for this report was obtained from the Ethics Committee of the Paul Doumer Hospital.

## CONSENT

Written informed consent was obtained from the patient for publication of this case report and the supporting images.

## Data Availability

The data supporting the findings of this study are available within the article.

## References

[1] Stevens DL , Bryant AE , Goldstein EJ . Necrotizing soft tissue infections. Infect Dis Clin North Am. 2021;35(1):135‐155. doi:10.1016/j.idc.2020.10.004 33303335

[2] Chen LL , Fasolka B , Treacy C . Necrotizing fasciitis: a comprehensive review. Nursing. 2020;50(9):34‐40. doi:10.1097/01.NURSE.0000694752.85118.62 PMC8828282 32826674

[3] Nelson GE , Pondo T , Toews KA , et al. Epidemiology of invasive group a streptococcal infections in the United States, 2005‐2012. Clin Infect Dis. 2016;63(4):478‐486. doi:10.1093/cid/ciw248 27105747 PMC5776658

[4] Misiakos EP , Bagias G , Patapis P , Sotiropoulos D , Kanavidis P , Machairas A . Current concepts in the management of necrotizing fasciitis. Front Surg. 2014;1:36. doi:10.3389/fsurg.2014.00036 25593960 PMC4286984

[5] Shiroff AM , Herlitz GN , Gracias VH . Necrotizing soft tissue infections. J Intensive Care Med. 2014;29(3):138‐144. doi:10.1177/0885066612463680 23753218

[6] Abdalla TSA , Grotelüschen R , Abdalla ASA , Melling N , Izbicki JR , Bachmann K . Prognostic factors for intraoperative detection of necrotizing fasciitis in severe soft tissue infections. PLoS ONE. 2023;18(5):e0285048. doi:10.1371/journal.pone.0285048 37134092 PMC10156062

[7] Stevens DL , Bisno AL , Chambers HF , et al. Practice guidelines for the diagnosis and management of skin and soft tissue infections: 2014 update by the Infectious Diseases Society of America. Clin Infect Dis. 2014;59(2):e10‐e52. doi:10.1093/cid/ciu444 24973422

[8] Sawai J , Hasegawa T , Kamimura T , et al. Growth phase‐dependent effect of clindamycin on production of exoproteins by *Streptococcus pyogenes* . Antimicrob Agents Chemother. 2007;51(2):461‐467. doi:10.1128/AAC.00539-06 17101685 PMC1797754

[9] Ditsios K , Chitas K , Christidis P , Charatsis K , Katsimentzas T , Papadopoulos P . Necrotizing fasciitis of the upper extremity – a review. Orthop Rev (Pavia). 2022;14(3):35320. doi:10.52965/001c.35320 36034724 PMC9404292

[10] Moore PJ , Foster L . Cost benefits of two dressings in the management of surgical wounds. Br J Nurs. 2000;9(17):1128‐1132. doi:10.12968/bjon.2000.9.17.5464 11868167

[11] Bowler PG , Jones SA , Davies BJ , Coyle E . Infection control properties of some wound dressings. J Wound Care. 1999;8(10):499‐502. doi:10.12968/jowc.1999.8.10.26356 10827654

